# Case of Divided Trachea

**Published:** 1802-08-01

**Authors:** S. Goe

**Affiliations:** Surgeon, of Louth


### *Case of divided Trachea; communicated by* Mr. S. Goe, *Surgeon, of Louth.*

ISABELLA HARE, about twenty-eight years of age, on the 20th of laſt September, in a ſtate of mental derangement, made an attempt to deſtroy herſelf by cutting her throat. She was in the ſervice of Mr. Naul, of this town, and not long before had abruptly left her place in a fit of melancholy; from this lowneſs ſhe gradually recovered, and was, at her own requeſt, taken again into her former ſervice, where ſhe had before experienced every indulgence. She however ſuddenly relapſed, and was miſſing ſoon after, early in the morning; diligent inquiries were immediately ſet on foot to find her, but to no purpoſe, as ſhe was not ſeen, or heard of, before the following morning about 9 o'clock, when ſhe was diſcovered in the chamber of a warehouſe, with her throat cut. In this ſituation I was ſent for, and found her ſenſeleſs, and almoſt without life, having no perceptible pulſe at the wriſt, very cold, and a convulſive ſtare with the eyes; reſpiration was performed at the wound of the trachea, but ſo laborious that the noiſe in taking her breath firſt led to the diſcovery. There being no hæmorrhage, a pledget of lint was applied over the wound; and being wrapt in a blanket, ſhe was conveyed to the workhouſe, which is only a ſmall diſtance from the place where ſhe was found. As life appeared ſo nearly exhauſted, ſome of the means recommended in ſuſpended animation were firſt made uſe of, ſuch as frictions, flannels, and warmth gradually applied, which in the courſe of an hour had the effect of reſtoring the circulation to the extremities; by this time ſhe alſo recovered her ſenſes, and ſhewed ſigns of contrition, but was not able to ſpeak. Unfortunately, none of my medical aſſociates were at home to aſſiſt me with their advice in this unfortunate caſe, and I was obliged to proceed to dreſs the wound by myſelf. A complete diviſion of the trachea was tranſverſely made, immediately under that prominent part of the throat called by anatomiſts, pomum Adami; the larynx appeared much leſſened in its diameter, and the two portions did not recede leſs than an inch and a half from each other; the muſcles on each ſide were partially divided, and the wound from one extremity to the other meaſured about three inches; a frothy mucus mixed with blood was brought up at every expiration. In this ſtate I gave her ſome liquids, to aſcertain the ſafety of the gula, and found they paſſed without any other inconvenience than the irritation occaſioned by preſſure on the injured parts. As ſhe was incapable of uttering the leaſt noiſe, I ſuſpected the entire loſs of voice aroſe from from an injury done to the branches of the recurrent nerves; but in this I was probably miftaken, the edges were jagged from the bluntnefs of the knife, and were become white from the length of time that had elapfed after the injury.

My next ftep was to bring the divided parts into contact; here I was much perplexed, as Mr. B. Bell recommends the futures to be made only in the furrounding integuments; but the inferior portion had receded fo low; I thought there was no chance left to bring them together but by a future through the very fubftance of the trachea. I accordingly paffed my finger and thumb into the wound, to bring up the retracted part, and, by a future made with one of my largeft needles, fucceeded in keeping the cut furfaces much nearer; another future was made upon the integuments. The lips of the wound were, by thefe means clofed, and the air prevented from efcaping externally. I then refolved to proceed no farther till I had witneffed the effects of this treatment, and had taken the opinion of another furgeon. After the wound was dreffed up, and the chin brought to reft upon the fternum, fhe immediately recovered her voice, and requefted a clergyman to pray with her. Broth glyfters and nourifhment, in a liquid form, were ordered, with an anodyne at bed-time. The next morning I was met by Mr. King, who, as furgeon to the poor, was firft applied to; fhe had paffed a good night, without any confiderable irritation of the trachea; it was then agreed to let the two former futures alone, and make a third through the integuments only, to keep the parts more firmly united, in hopes the wound might heal by the firft intention. In this expectation we were difappointed, as on the fixth or feventh day all the ftitches loofened, and the air again made its efcape from a pretty confiderable aperture; we, however, made another attempt to unite the edges of the wound by making three frefh futures upon the parts moft free from inflammation; and although a fufficient hold was taken of the found fkin, in about four days they broke through in the fame manner, and the air again made its efcape. Under thefe circumftances there appeared no refources left, but trufting to the efforts of Nature, with a proper attention to fupport the ftrength, and keep the wound in a favorable pofition. In the courfe of a few days, granulations fprung up rapidly from both furfaces of the fore, and in lefs than a month they had united through nearly the whole extent of the wound; a fmall circular opening, remained upon the fore part of the trachea for fome weeks afterwards, and was with difficulty clofed, from the high degree of inflammation leaving the integuments very rigid and unyielding; but at length it was healed with the affiftance of fticking plafter. Whilft the aperture remained, it was found

 neceffary neceffary to keep a comprefs upon the part, conftantly to confine the air in the larynx, as the leaft efcape took away the voice. It may be obferved, that although fhe had regained her appetite, and could fwallow folids without inconvenience, yet her acceffion of ftrength was far from keeping pace with the ingefta, as at this time fhe was fcarcely able to ftand by herfelf. How far this circumftance was owing to the ftate of the larynx, or to the previous diforder of the mind, I am unable to determine, but am inclined to think the opening in the wind-pipe by fome means influenced the fyftem in laying the foundation of great debility.

Three things occurred in this cafe, which induced me to think it might not be altogether unworthy the notice of other practitioners. Firft, that a complete divifion of the trachea, unaccompanied with a wound of the large blood-veffels, or œfophagus, is not always mortal, as confidered by Heifter, and fome other old furgeons. Secondly, that when the edges of the divided trachea have receded to a great diftance, it may be fafe to pafs a future directly through its fubftance, without creating an alarming cough and irritation. Thirdly, that the healing powers of Nature will effect a cure in fome inftances, under circumftances extremely difcouraging to the practitioner.

*Louth, December* 27, 1801.

